# Sinus Venosus Atrial Septal Defect as a Cause of Palpitations and Dyspnea in an Adult: A Diagnostic Imaging Challenge

**DOI:** 10.1155/2015/128462

**Published:** 2015-01-29

**Authors:** Michael S. Donovan, David Kassop, Robert A. Liotta, Edward A. Hulten

**Affiliations:** ^1^Cardiology Department, Walter Reed National Military Medical Center, 8901 Wisconsin Avenue, Bethesda, MD 20889, USA; ^2^Radiology Department, Walter Reed National Military Medical Center, 8901 Wisconsin Avenue, Bethesda, MD 20889, USA

## Abstract

Sinus venosus atrial septal defects (SV-ASD) have nonspecific clinical presentations and represent a diagnostic imaging challenge. Transthoracic echocardiography (TTE) remains the initial diagnostic imaging modality. However, detection rates have been as low as 12%. Transesophageal echocardiography (TEE) improves diagnostic accuracy though it may not detect commonly associated partial anomalous pulmonary venous return (PAPVR). Cardiac magnetic resonance (CMR) imaging provides a noninvasive, highly sensitive and specific imaging modality of SV-ASD. We describe a case of an adult male with exercise-induced, paroxysmal supraventricular tachycardia who presented with palpitations and dyspnea. Despite nondiagnostic imaging results on TTE, CMR proved to be instrumental in visualizing a hemodynamically significant SV-ASD with PAPVR that ultimately led to surgical correction.

## 1. Introduction

Sinus venosus atrial septal defects (SV-ASD) involve the insertion of the superior or inferior vena cava and account for 2–12% of all atrial communication abnormalities [1]. The defect does not involve the embryologic atrial septum but most often affects the superior vena cava at the junction of the right atrium. There are at least one or more anomalous connections involving the right pulmonary vein(s) to the superior or inferior vena cava or the right atrium. Most patients present with exertional dyspnea, though the age at onset of symptoms is highly variable. Other clinical manifestations include paradoxical embolism and recurrent pulmonary infections. Arrhythmias have been observed when the degree of shunt has resulted in significant atrial dilation, typically during the fourth decade of life [2]. Comprehensive diagnostic evaluation often requires more advanced imaging to include transesophageal echocardiography (TEE), cardiac magnetic resonance (CMR) imaging, and/or electrocardiography- (ECG-) gated cardiac computed tomography (CT).

## 2. Case Presentation

**Figure 1:**
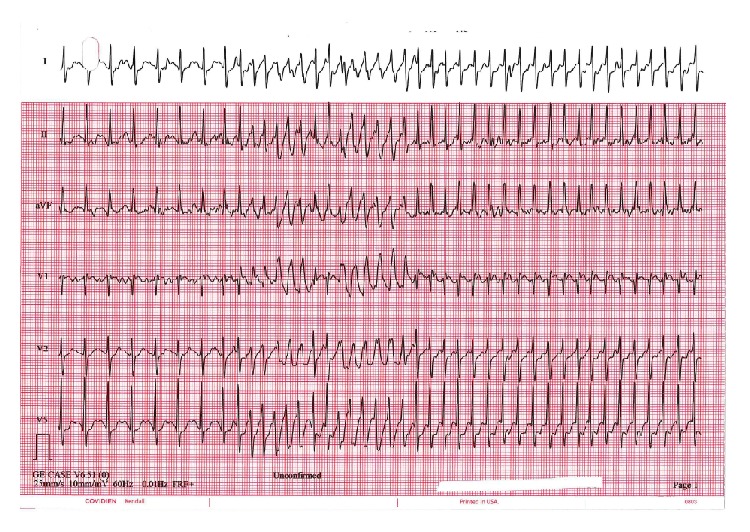
Electrocardiogram obtained during exercise stress test demonstrating sinus tachycardia at approximately 155 beats per minute (bpm). There is an abrupt onset of supraventricular tachycardia with intermittent aberrant conduction and a ventricular rate of 290 bpm. Standard paper speed is 25 millimeters per second, and amplitude is 10 millimeters per millivolt.

A 59-year-old male with a past medical history of hypertension presented with a two-month history of exertional palpitations, lightheadedness, and dyspnea. His resting ECG was normal. On exercise stress testing, the patient developed supraventricular tachycardia (SVT) with a ventricular rate of 292 beats per minute with reproducible symptoms (Figure 1). Transthoracic echocardiography (TTE) demonstrated mild right atrial and ventricular enlargement and a dilated pulmonary artery (PA) with an estimated pulmonary arterial systolic pressure of 45–50 mmHg. Right ventricular systolic function was normal. An agitated saline microbubble study was positive with near simultaneous observation of microbubbles in the right and left atria consistent with intracardiac shunting. There was no additional evidence of an atrial septal defect to include color-flow Doppler interrogation. Shunt calculation by TTE measurements revealed a pulmonary-to-systemic blood flow ratio (Qp/Qs) of 1.7.

A right heart catheterization (RHC) revealed severe pulmonary hypertension with a mean pulmonary arterial pressure of 35 mmHg and an elevated pulmonary arterial oxygen saturation of 81%. Left heart catheterization with selective coronary angiography demonstrated no evidence of coronary artery disease. The cardiac catheterizations were performed prior to any suspicion for intracardiac shunting, immediately following the diagnosis of supraventricular tachycardia. A full oxygen saturation evaluation on RHC was not performed for unclear reasons. Transesophageal echocardiography was also not performed at the request of the patient; CMR imaging was offered as an alternative imaging option.

**Figure 2:**
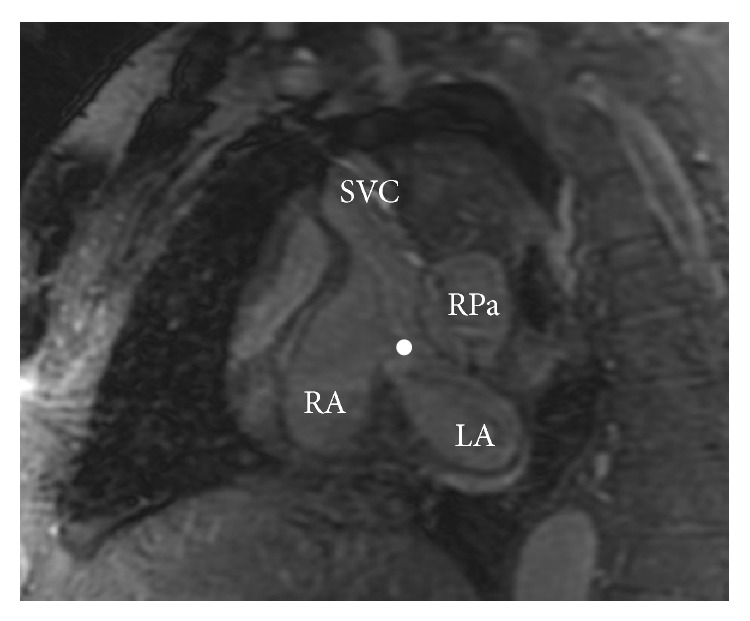
Sagittal 3-dimensional steady state free precession (SSFP) magnetic resonance image demonstrating a large sinus venosus defect (circle). SVC: superior vena cava; RPa: right pulmonary artery; RA: right atrium; LA: left atrium.

**Figure 3:**
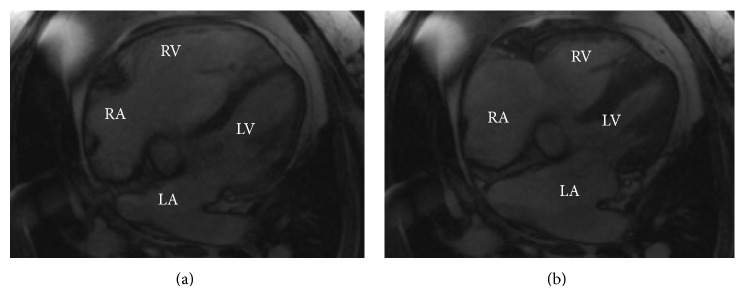
Long axis cardiac magnetic resonance imaging prior to surgical correction. Panel (a) demonstrates severe right ventricular enlargement during end-diastole of the cardiac cycle. Panel (b) demonstrates severe right atrial enlargement during end-systole of the cardiac cycle. RA: right atrium; RV: right ventricle; LA: left atrium; LV: left ventricle.

**Figure 4:**
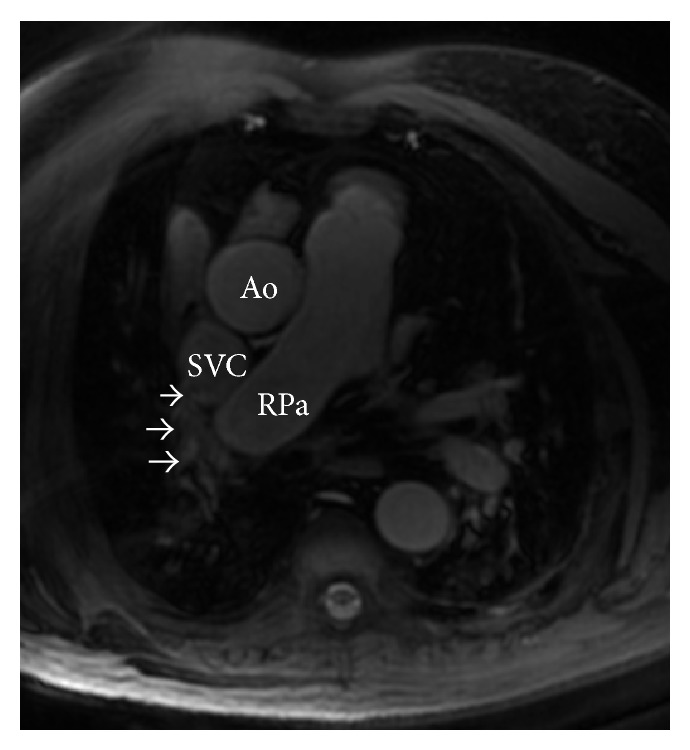
Axial 3-dimensional steady state free precession (SSFP) magnetic resonance image demonstrating anomalous pulmonary venous return of the right superior pulmonary vein into the superior vena cava. Right upper lobe partial anomalous pulmonary venous return (arrows). SVC: superior vena cava; RPa: right pulmonary artery; Ao: aorta.

On CMR, a large SV-ASD was observed with severe right atrial and ventricular enlargement (Figures 2 and 3). The PA was dilated at 4.2 cm. Additionally, a partial right-upper lobe anomalous pulmonary venous connection was identified (Figure 4) with hemodynamically significant shunting (Qp/Qs 2.2).

**Figure 5:**
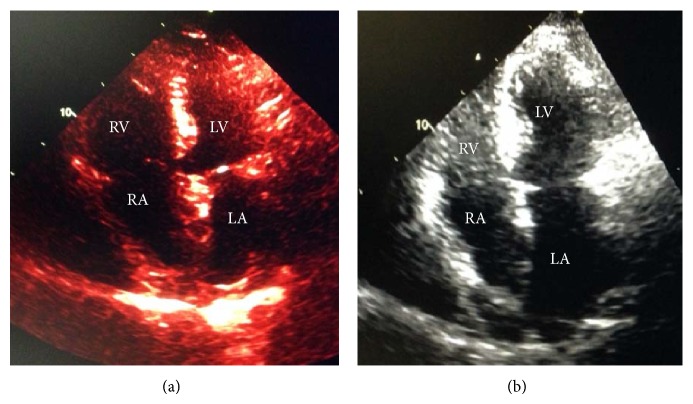
Two-dimensional transthoracic echocardiography in the apical four-chamber view. Panel (a) was obtained prior to surgical correction and demonstrates significant right atrial and ventricular enlargement. Panel (b) was obtained six months following surgical correction and demonstrates normalization of the right atrial and ventricular sizes. RA: right atrium; RV: right ventricle; LA: left atrium; LV: left ventricle.

The patient subsequently underwent successful surgical correction of the SV-ASD and PAPVR with an initial resolution of symptoms. At a routine six-month follow-up, TTE demonstrated normal right atrial and ventricular size and function (Figure 5). There was mild residual pulmonary hypertension, with an estimated PASP of 35–40 mmHg. The patient however endorsed recurrent episodes of palpitations. Ambulatory monitoring revealed frequent premature atrial contractions and short runs of paroxysmal SVT, lasting less than five seconds. Although a trial of beta blocker therapy failed to improve symptoms, the patient was initiated on sotalol with successful symptom control and suppression of atrial ectopy.

## 3. Discussion

Sinus venosus atrial septal defects have nonspecific clinical presentations with a variable age of onset and should always be considered in the setting of unexplained right atrial and ventricular enlargement. Advanced imaging modalities may be required to facilitate a diagnosis given the location of the defect and their associated anomalous vascular connection(s). Transthoracic echocardiography remains first line in diagnostic evaluation though detection rates have been reported as low as 12 to 44% with the highest success rate utilizing the subcostal view [3, 4].

Transesophageal echocardiography has been shown to increase the detection of SV-ASD and the presence of PAPVR [5, 6]. In a retrospective study comparing TEE with surgery, 10 additional anomalous venous connections were identified at the time of surgery that were not visualized during the TEE [5]. ECG-gated cardiac CT has been shown to accurately detect anomalous pulmonary venous connections [7]. This particular modality, while useful, lacks the ability to calculate shunt fractions and exposes the patient to nephrotoxic contrast agents and ionizing radiation.

Cardiac magnetic resonance imaging has been shown to accurately detect SV-ASD and identify anomalous pulmonary venous connections. In a small retrospective review of patients without a conclusive diagnosis despite TEE, CMR was able to successfully identify a sinus venosus defect in 7 of 11 patients [6]. This modality has the additional benefit of cardiac chamber size quantification, ventricular function assessment, and shunt fraction calculation, while sparing the patient from an invasive procedure or exposure to ionizing radiation [3, 8]. Furthermore, successful imaging has been achieved utilizing protocols that do not require gadolinium contrast agents [6].

A comprehensive approach must be undertaken when evaluating a patient with unexplained right heart dilation. Routine initial diagnostic imaging techniques may not prove sufficient to establish a diagnosis particularly in the case of sinus venosus defects given the location and associated anomalies. The collective data obtained by CMR provides additive diagnostic value when determining future management to include surgical correction, particularly when other methods have not established a diagnosis.
